# Advanced Demodulation in Distributed Fiber Optic Sensing: A Review of Backscattering and UWFBG-Based Technologies

**DOI:** 10.3390/s26051674

**Published:** 2026-03-06

**Authors:** Yiming Wang, Liang Zhang, Canyang Sun, Changjia Wang, Xin Gui, Xuelei Fu, Zhengying Li

**Affiliations:** 1 School of Information Engineering, Wuhan University of Technology, Wuhan 430062, China; wangyiming@whut.edu.cn (Y.W.); z.liang@whut.edu.cn (L.Z.); sun_canyang@whut.edu.cn (C.S.); wangchangjia@whut.edu.cn (C.W.); xlfu@whut.edu.cn (X.F.); 2 National Engineering Research Center of Fiber Optic Sensing Technology and Networks, Wuhan University of Technology, Wuhan 430062, China; guixin@whut.edu.cn

**Keywords:** distributed fiber optic sensing, ultra-weak fiber Bragg grating, optical fiber backscattering, demodulation technology

## Abstract

Distributed fiber optic sensing (DFOS) has emerged as a critical technology for structural health monitoring of large-scale infrastructure, offering unique advantages in terms of coverage and environmental adaptability. This review presents a comprehensive analysis of the two dominant technical routes: fully distributed sensing based on intrinsic backscattering and massive-capacity sensing based on ultra-weak fiber Bragg grating (UWFBG) networks. For backscattering-based systems—encompassing Raman, Brillouin, and Rayleigh scattering—the inherent trade-offs among signal-to-noise ratio (SNR), spatial resolution, and sensing range constitute major performance bottlenecks. This review systematically summarizes advanced demodulation and signal processing strategies designed to overcome these physical barriers, including pulse coding sequences, chaotic laser compressed correlation, and deep learning-enhanced noise reduction algorithms. In parallel, for UWFBG-based technologies, the evolution from traditional multiple-point fiber Bragg grating (FBG) array to quasi-distributed and fully distributed UWFBG network is discussed. This review highlights key breakthroughs in achieving high spatial resolution and high-speed interrogation through hybrid multiplexing, aliased spectrum reconstruction, and dispersion-based demodulation techniques. By synthesizing recent advances in modulation schemes, detection hardware, and algorithmic processing, this paper outlines the trajectory of DFOS technologies toward high-precision, long-distance, and real-time sensing networking.

## 1. Introduction

Large-scale critical infrastructure, such as long-distance oil and gas pipelines, high-speed railway tunnels, power transmission grids, large-span bridges, buildings, and constructions, constitutes the lifeline of modern society [1,2,3,4,5,6,7]. Ensuring the structural integrity and operational safety of these assets is of paramount importance. Traditional point-based sensors (e.g., strain gauges and accelerometers) often suffer from limited coverage, electromagnetic interference, and difficulty in cabling for long-distance applications, making them insufficient for monitoring infrastructure that spans tens or hundreds of kilometers [8,9,10]. Optical sensing technologies span from plasmonic and resonant on-chip sensors for highly sensitive localized detection to fiber-optic platforms enabling long-distance, large-scale distributed monitoring, forming a multi-scale sensing framework that bridges micro–nano devices and infrastructure-scale applications [11,12,13]. In this context, DFOS has emerged as a revolutionary technology. Acting as a “nervous system” for infrastructure, DFOS utilizes the optical fiber itself as the sensing medium to capture external physical fields—such as temperature, strain, and vibration—continuously along the entire fiber length [14,15,16].

The development of DFOS can be traced back to the concept of optical time domain reflectometry (OTDR), introduced in 1977, initially for fiber fault detection [17]. Over the past few decades, technology has evolved from simple attenuation measurement to sophisticated sensing systems capable of resolving phase, frequency, and polarization changes. Depending on the sensing mechanism, DFOS is generally categorized into two main streams: scattering-based fully distributed sensing [18] and grating-based quasi-distributed sensing [13].

The first category, fully distributed sensing, relies on the intrinsic scattering effects within the silica fiber core. Rayleigh scattering is widely used for vibration and acoustic sensing, such as phase-sensitive optical time domain reflectometer (φ-OTDR) or distributed acoustic sensing (DAS) [19,20]; Brillouin scattering provides temperature and strain information (e.g., Brillouin optical time domain reflectometry) [21,22]; and Raman scattering is the standard for distributed temperature sensing (DTS) [23]. The key advantage of these technologies is the ability to use standard single-mode telecommunication fibers without any modification, achieving sensing ranges of up to 100 km. However, they face fundamental physical limits. The intrinsic backscattering coefficients are extremely low (typically below –80 dB/m), leading to weak signal intensity. Consequently, these systems suffer from an inherent trade-off among SNR, spatial resolution, and sensing range [24,25,26]. For instance, narrowing the optical pulse to improve spatial resolution inevitably reduces the pulse energy, thereby degrading the SNR and limiting the sensing distance. Here, the spatial resolution is uniformly defined as the minimum distance at which the system can distinguish two adjacent disturbance events along the optical fiber direction. Its essence corresponds to the effective width of the system’s spatial response function. The sensing distance is uniformly defined as the maximum effective measurement length under the condition of meeting specific measurement uncertainty or signal-to-noise ratio threshold requirements, rather than being solely determined by the criterion of “being able to detect the signal”. Generally, it can be defined as the effective measurement length of the optical fiber. Measurement accuracy is the key sensing indicator of the system, and it is used to describe the deviation between the measured value and the true value. It can be determined by the standard deviation or uncertainty of the measured value. Resolution refers to the system’s uncertainty, which describes the system’s ability to distinguish minute changes. It is usually defined as the standard deviation of the measured value under constant conditions [12,27].

The second category is based on fiber Bragg grating (FBG) arrays. Traditional FBG sensors offer high SNR and multi-parameter precision but were historically limited to quasi-distributed applications due to the complexity of multiplexing and the limited number of gratings (typically fewer than 100) that could be inscribed on a single fiber [28,29]. However, recent advancements in online writing technology have enabled the mass production of ultra-weak fiber Bragg grating (UWFBG) networks. By reducing the reflectivity to an extremely low level (e.g., −40 dB to −50 dB), thousands or even tens of thousands of gratings can be integrated into a single fiber with negligible crosstalk, forming a continuous, massive-capacity sensing network that rivals the density of fully distributed systems while retaining the high precision of gratings [30,31,32].

Despite these advances, the demand for higher spatial resolution (millimeter level), longer distances (>50 km), and real-time response (>kHz) in modern engineering applications places immense pressure on signal demodulation [33]. Simple intensity detection is no longer sufficient. Advanced demodulation has thus become the core driver of DFOS performance enhancement. This involves hardware innovations—such as coherent detection, chaotic lasers, and frequency-swept linearization—and, more importantly, software breakthroughs. Advanced signal processing techniques, including pulse coding sequences (e.g., Golay and Simplex), compressed sensing, image denoising algorithms, and deep learning, are being extensively investigated to break the physical limitations [34,35,36,37].

This review provides a comprehensive analysis of advanced demodulation and performance enhancement technologies for both backscattering-based and UWFBG-based systems. Section 2 focuses on fully distributed scattering information networking, detailing improvements in Raman optical time domain reflectometry (ROTDR), Brillouin optical time domain reflectometry (BOTDR), φ-OTDR, and optical frequency domain reflectometry (OFDR) through pulse coding, chaotic correlation, and AI-driven noise reduction. Section 3 discusses the evolution of UWFBG-based networking, highlighting the transition to UWFBG arrays and the advanced φ-OTDR/OTDR/OFDR interrogation schemes designed for high-capacity, high-speed, and high-resolution demodulation. Finally, this review summarizes the current state of the art and outlines future trends toward intelligent, integrated, and ultra-long-distance sensing networks.

In addition to summarizing representative sensing principles and interrogation architectures, this review further adopts a demodulation-oriented perspective to interpret the recent evolution of DFOS networks. Specifically, we emphasize that the progress of both backscattering-based and UWFBG-based DFOS is reflected not only in the diversification of sensing mechanisms but also in the continuous refinement of demodulation strategies for balancing multiple coupled performance constraints in practical systems. These constraints typically include SNR, spatial resolution, sensing range, demodulation speed, stability, and robustness under complex field conditions. From this perspective, different demodulation methods can be understood not merely as isolated algorithmic or instrumental improvements but as system-level approaches for reshaping the performance boundary of DFOS under different application requirements. Therefore, beyond a categorical review of recent studies, this paper also highlights the underlying evolution logic of demodulation technologies, namely, the transition from single-metric optimization toward coordinated optimization of optical architecture, signal processing, and application-oriented system performance.

## 2. Backscattering-Based DFOS Networks

Distributed optical scattering sensing networking is usually based on the inherent backscattering principle of optical fibers combined with the demodulation technology of scattering sensing. Its core technology is based on three optical scattering mechanisms: Raman scattering for temperature measurement, Brillouin scattering responsive to both temperature and strain, and Rayleigh scattering. To achieve the demodulation of the variations in the backscattered optical signals, two primary technological approaches are employed: OTDR and OFDR.

This section follows a consistent problem–solution narrative within each sensing mechanism. For Raman-, Brillouin-, and Rayleigh-based scattering sensing, we first summarize the fundamental performance limitations and then categorize representative demodulation advances into three recurring solution families: (i) hardware-level modulation/interrogation improvements, (ii) coding/compression strategies, and (iii) algorithmic processing for compensation and denoising. In addition, brief transition notes are added to connect OTDR- and OFDR-based discussions for the same sensing mechanism, and a concise summary table is provided at the end of each major subsection to highlight key advances and remaining gaps.

### 2.1. OTDR Demodulation Technology

OTDR is a technology that utilizes the propagation and reflection characteristics of light pulses in optical fibers to locate and quantify physical quantities on the optical fiber line, as well as the properties of the fiber itself. By emitting a light pulse into the optical fiber, information from the backscattered light and the return time are analyzed to obtain the spatial distribution information of the entire optical fiber.

In traditional OTDR optical detection systems, the trade-off among signal-to-noise ratio, spatial resolution, and detection range is a common, physically principle-based inherent contradiction. Fundamentally, this trade-off is aimed at enhancing one performance metric, often at the expense of another. The pursuit of high spatial resolution not only relies on extremely narrow pulses but also forces the system to adopt detectors and circuits with extremely high sensitivity and gain, which directly leads to a factorial increase in cost. Such a design, while enhancing resolution, inevitably weakens the signal-to-noise ratio due to the sharp reduction in single-pulse energy. To maintain necessary measurement accuracy under low signal-to-noise ratios, the effective detection range of the system has to be significantly narrowed. Conversely, if one wants to improve the signal-to-noise ratio and expand the detection range, the pulse width or energy needs to be increased, which will directly lead to a deterioration in spatial resolution. For example, in a pulse flight time-based detection system, to ensure a sufficiently strong echo signal, a wider pulse needs to be used. However, this directly leads to a decline in the system’s ability to distinguish spatially adjacent targets, that is, a deterioration in spatial resolution. Therefore, the optimization of any single performance metric often comes at the expense of other metrics. Current technological developments, such as advanced signal processing algorithms and multi-sensor fusion schemes, are striving to break through or alleviate this fundamental limitation in specific application scenarios, in order to seek a better overall performance balance and achieve better comprehensive performance in specific application scenarios [12].

#### 2.1.1. Raman Optical Time Domain Reflectometry

Distributed optical fiber sensing based on Raman scattering detects temperature changes by measuring the intensity ratio of the anti-Stokes light and the Stokes light in the optical fiber. In the ROTDR scheme combined with OTDR technology, the intensity information of Raman scattering light is utilized for time domain networking. By analyzing the time delay information of Raman scattering light, the scattered signal can be separated and spatially located in the time domain, thereby achieving long-distance temperature distribution monitoring. However, compared with Rayleigh scattering and Brillouin scattering, Raman scattering has the weakest intensity and a lower system SNR, which limits the improvement of the sensing performance. To enhance the SNR, traditional methods often increase the power of the pulsed laser, but excessive power will induce nonlinear effects, which instead affect the accuracy of temperature demodulation. On the other hand, compressing the pulse width of the light source can improve the spatial resolution of the system, but this optimization will simultaneously lead to a decrease in the incident light power, thereby limiting the maximum sensing distance of the system. To overcome these limitations, recent research has primarily focused on three optimization strategies.

**Figure 1:**
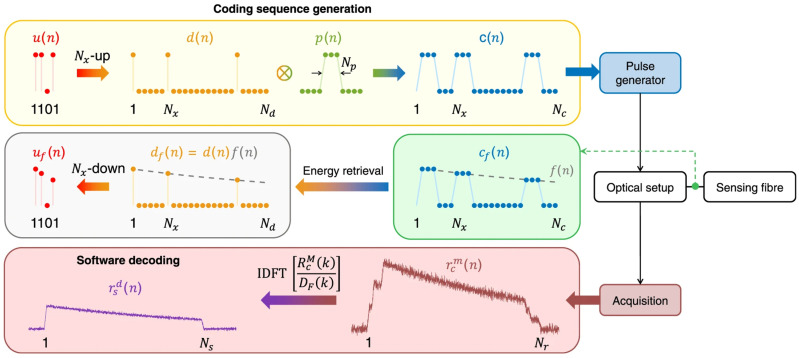
Principle of the genetic-optimized aperiodic coding and decoding process [35].

**Figure 2:**
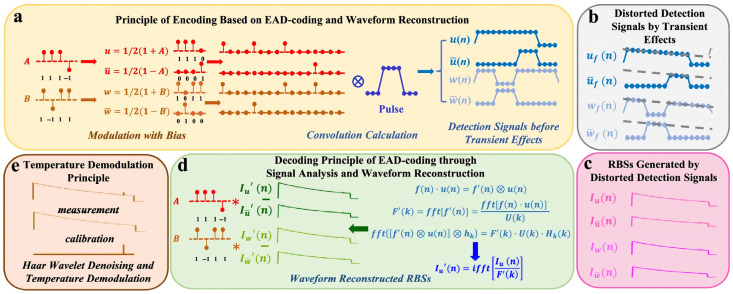
Physics principle of EAD coding and waveform reconstruction scheme for Raman distributed optical fiber sensing: (**a**) Principle of encoding based on EAD coding and waveform reconstruction. (**b**) Influence of transient effects on detection signals. (**c**) RBSs generated by four distorted detection signals in sensing fiber. (**d**) Decoding principle of EAD coding through signal analysis and waveform reconstruction, where * is the correlation operator. (**e**) Temperature demodulation principle [27].

By encoding and modulating the detection pulse source through the pulse coding scheme and designing the corresponding decoding process in the demodulation system, the SNR and dynamic range can be improved without increasing the power of a single pulse. In 2011, Soto et al. used the low repetition rate quasi-periodic pulse coding technology to achieve a spatial resolution of 1 m on a 26 km single-mode optical fiber, with a temperature accuracy of 3 °C [38]. Vazquez et al. used the cyclic pseudo-random pulse sequence coding technology to increase the SNR by 11 times and achieve a spatial resolution of 2 m and a temperature resolution of 1.5 °C [39]. Sun et al. proposed in 2020 to use a single-sequence genetic-optimized aperiodic code to encode the optical signal, and through fast demodulation processing, they obtained a temperature measurement accuracy of 0.5 °C at a sensing distance of 40 km [35]. Figure 1 illustrates the principle of the genetic-optimized aperiodic code and decoding process. In Raman-based distributed optical fiber sensing systems, early implementations were fundamentally constrained by the intrinsic trade-off between signal-to-noise ratio (SNR) and sensing range, with performance degrading significantly beyond 30 km. In response to the inherent low SNR limitation in long-distance sensing, Zhang et al. propose a paradigm that combines enhanced anti-distortion (EAD) coding processing, advanced Raman scattering waveform reconstruction preprocessing, and Haar wavelet denoising to transcend this physical limitation. The experimental demonstration achieves performance metrics of 70.0 km sensing distance with 1.58 m spatial resolution, while maintaining 0.91 °C measurement accuracy and 5.39 °C temperature resolution [27]. Figure 2 illustrates the physics principle of EAD coding and waveform reconstruction scheme for Raman distributed optical fiber sensing. The sequences A and B represent a pair of Golay complementary sequences. Using long sequences with high peak power can induce backward stimulated Raman scattering (SRS), disrupting the linear superposition of spontaneous Raman scattering responses required for reliable decoding, and causing significant temperature estimation errors. To address this issue, Li et al. proposes a compensation method. Experimental results over a 24 km fiber using 130-bit genetic-optimized codes and 1 W peak power pulses demonstrate that the method effectively suppresses SRS-induced distortions without compromising the spatial resolution or the SNR improvement provided by the coding gain [40].

**Figure 3:**
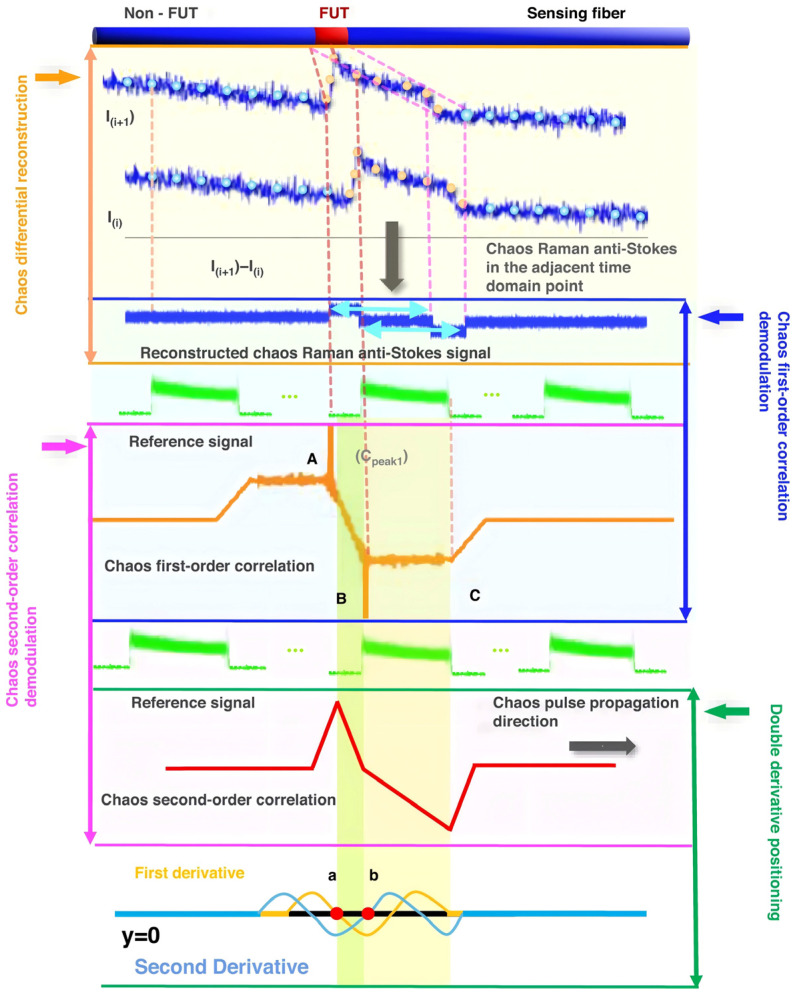
Principle of chaos differential reconstruction and double correlation demodulation in the chaos Raman distributed optical fiber sensing scheme. Position A represents the positive correlation peak generated by the chaotic first-order correlation demodulation method, while position B represents the negative correlation peak. Position C corresponds to the end point where the chaotic second-order correlation demodulation signal drops. Position a is the point where the first-order derivative of the chaotic second-order correlation demodulation signal is zero, corresponding to the starting position of the FUT region, and Position b is the nearest point where the second-order derivative is zero, corresponding to the ending position of the FUT region [44].

By compressing the relevant demodulation schemes, the detected pulse source with random fluctuation characteristics is demodulated with the differential reconstructed Raman anti-Stokes optical signal, resulting in a higher SNR and spatial resolution that is not limited by the pulse width of the traditional optical time domain reflection technology. In 2021, Li and Zhang et al. [41] first proposed this compressed correlation demodulation scheme, using an amplified spontaneous emission (ASE) source as the detection optical signal, and achieved temperature variations along the optical fiber line using the proposed compressed correlation demodulation scheme. In the simulation analysis, the spatial resolution reached 7.5 mm [41]. In the same year, they proposed a ROTDR system based on chaotic laser, using time domain differential reconstruction and short-scale time domain correlation compression technology. In the simulation analysis, they achieved a spatial resolution of 5 mm and a temperature sensitivity of 0.1 °C [42]. Subsequently, in their experiments, they used a chaotic laser with a 500 nanosecond pulse width, based on chaotic time domain differential reconstruction and chaotic correlation demodulation, and optimized the spatial resolution of the traditional optical time domain reflection system from 50 m to 0.3 m [43]. Through further improvement of the demodulation method, they used chaotic differential reconstruction and chaotic double correlation methods to detect the sensing distance and spatial resolution of the chaotic Raman distributed optical fiber sensing system. They achieved a spatial resolution of 10 cm and a sensing distance of 1.4 km, overcoming the physical limitation of the influence of wide pulse width on the sensing spatial resolution [44]. Figure 3 illustrates the principle of chaos differential reconstruction and double correlation demodulation in the chaos Raman distributed optical fiber sensing scheme.

**Figure 4:**
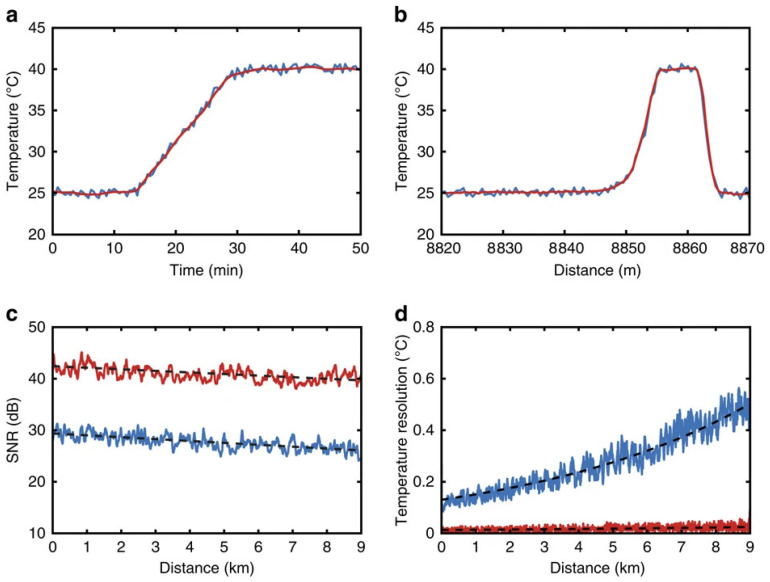
Impact of 2D image processing on 1D Raman traces. The performance of the sensor and the impact of 2D image denoising are evaluated while the temperature of 10 m at the end of the sensing fiber is slowly increased in time. Results obtained from the raw data (blue) are compared with the ones obtained from the denoised data using the 2D NLM method (red curve), in which a moving window of 21 consecutive traces is considered. Black dashed lines correspond to the respective linear fitting (in dB scale) of different signals. (**a**) Temporal evolution of the measured temperature at the hot spot location. This result shows that image processing induces no delay or distortion in the measured hot spot temperature. (**b**) Distributed temperature profile near the hot spot location, demonstrating that the denoising process produces no perceptible loss of spatial resolution. (**c**) SNR versus distance, validating an SNR enhancement of 13.6 dB at the end of the sensing range. (**d**) Temperature resolution versus distance, showing that the use of the 2D NLM method can improve the temperature uncertainty from 0.5 °C down to 0.022 °C [36].

By proposing a variety of digital signal processing algorithms, the noise could be efficiently reduced. The noise in distributed sensing systems based on Raman scattering stems primarily from Rayleigh scattering and other optical noises, as well as electrical noises generated by detectors and circuits. Through digital signal processing methods, the SNR of Raman scattering signals can be improved. In 2014, Wang et al. proposed an improved wavelet transform modulus maxima (WTMM) denoising method, which reduced the temperature error by approximately 2 °C at 30 °C and 60 °C, without reducing spatial resolution [45]. In 2018, Li and Zhang et al. [46] proposed and demonstrated an improved wavelet transform modulus maxima scheme combined with a dynamic difference denoising method for denoising processing. At a sensing distance of 10.4 km, the temperature measurement accuracy could reach 1.58 °C [46]. Soto et al. utilized the high similarity and redundancy contained in the multi-dimensional information measured by distributed temperature signals. Combined with traditional image and video processing, they achieved an unprecedented improvement in SNR and measurement contrast and could significantly enhance sensor performance by 100 times without the need for hardware modification [36]. Figure 4 illustrates the impact of 2D image processing on 1D Raman traces. Zhang and Tang et al. proposed and experimentally demonstrated a deep one-dimensional denoising convolutional neural network (1DDCNN) to improve the performance of Raman distributed temperature sensing (RDTS). Through multiple layers of convolution and nonlinear operations, any filtering effect can be achieved. At a length of 10 km for SMF, with an average measurement time of 1 s and a spatial resolution of 3 m, the temperature uncertainty decreased from 6.4 °C to 0.7 °C [47]. Yan et al. demonstrate a fast-non-local means (Fast-NLM) algorithm for performance improvement without modifying the hardware architecture of a conventional ROTDR system. The overall response time by using the method decreases from 27.9 s to 1.7 s, marking a 16-fold enhancement over traditional NLM denoising methodologies. An average temperature resolution is below 0.01 °C at the end of fiber. Compared with a conventional wavelet denoising algorithm, the root mean square error decreases from 1.02 °C to 0.42 °C [48].

#### 2.1.2. Brillouin Optical Time Domain Reflectometry

Distributed fiber sensing based on Brillouin scattering can measure the temperature or strain along the optical fiber by analyzing the frequency and power changes of the scattered light. The BOTDR technology, which combines with OTDR technology, has advantages such as single-ended incidence and full distributed sensing. However, on one hand, the peak power of the detection pulse is limited by factors such as the stimulated Brillouin scattering effect and the nonlinear effect. On the other hand, increasing the pulse width can improve the system SNR, but it will reduce the spatial resolution. To overcome these limitations, researchers mainly explore from two directions.

**Figure 5:**
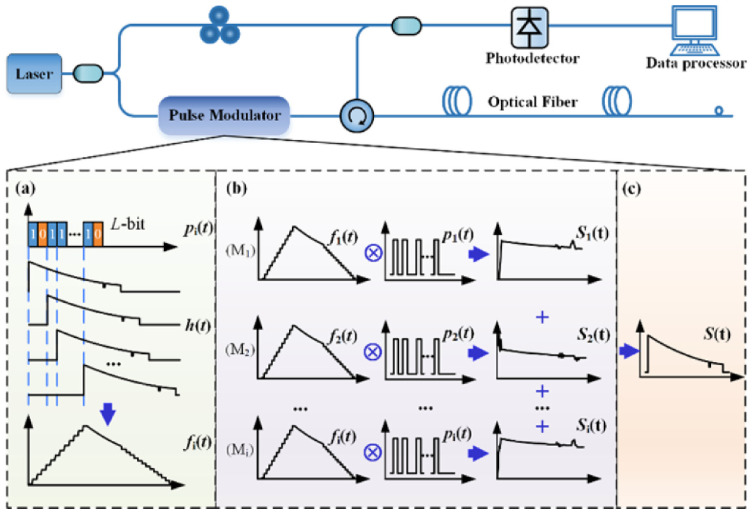
Decoding principle of random coding method in BOTDR sensor: (**a**) The backscattering signal formed by injecting encoded signals into the optical fiber. (**b**) The decoding process. (**c**) The reconstructed impulse response [52].

By encoding and modulating the detection pulse source through the pulse coding scheme and designing the corresponding decoding process in the demodulation system, the SNR and dynamic range can be improved without increasing the power of a single pulse. By increasing the number of pulses, the SNR and dynamic range can be enhanced. In 2017, Wang et al. first combined the Golay complementary coding scheme with FFT technology and applied it to the BOTDR system, proposing to pre-exhaust the dual-wavelength detection pulses to suppress the coding pulse distortion caused by the erbium-doped fiber amplifier (EDFA); they achieved a spatial resolution of 2 m on 10 km of single-mode fiber [49]. In 2018, Zan et al. utilized the differential cross-spectrum technology on 350 m of single-mode fiber to achieve a spatial resolution of 0.2 m and a Brillouin frequency shift (BFS) accuracy of 3.2 MHz [50]. In 2020, Zan et al. applied the Golay coding technology to the differential cross-spectrum Brillouin optical time domain reflection (DCS-BOTDR) system, achieving a spatial resolution of 40 cm with a single-mode fiber of 1280 m and a BFS accuracy of 3.47 MHz, with the BFS resolution optimizing from 4.28 MHz to 3.47 MHz as the coding length increased from 1 bit to 8 bits [51]. In 2022, Wang et al. obtained a coding gain of 11.93 dB by using a random coding scheme, reducing the uncertainty of the BFS of 4.93 km sensing fiber from 5.34 MHz to 0.38 MHz, and expanding the sensing range from 4.93 km to 64.76 km under the condition of 2 m spatial resolution and root mean square error (RMSE) ≤ 3 MHz [52]. Figure 5 illustrates the decoding principle of a random coding method in a BOTDR sensor. In 2022, Zan et al. used the DCS-BOTDR technology with optimized long pulse parameters on 1.2 km of single-mode fiber to achieve a BFS resolution of approximately 5.3 MHz; on 4 km of single-mode fiber, using a combination of 18 ns long pulses and 4 ns short pulses, they achieved a spatial resolution of 40 cm and a temperature measurement accuracy of 4 °C [53]. In 2025, Zhang et al. applied the single-channel coding demodulation scheme to the BOTDR system with short-time Fourier transform processing, achieving a total measurement time of 60 s, a spatial resolution of 4.57 m, and a BFS accuracy of 1.34 MHz in the measurement of 80.3 km of single-mode fiber (two sections of G.652.D optical fibers connected in series), while obtaining a 7.2 dB improvement in SNR [54].

**Figure 6:**
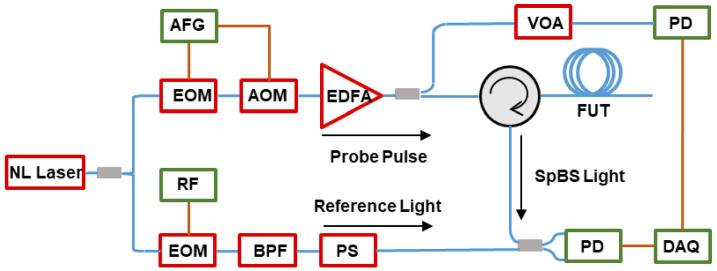
BOTDR system. NL Laser: narrow linewidth laser; EOM: electro-optic modulator; AOM: acoustic optical modulator; EDFA: erbium-doped fiber amplifier; BPF: bandpass filter; PS: polarization scrambler; PD: photodetector; DAQ: data acquisition card; VOA: variable optical attenuator; AFG: Arbitrary Function Generator; RF: Radio Frequency; FUT: fiber under test [55].

By optimizing the performance of the BOTDR through post-processing techniques, without altering the hardware structure or increasing the pump single-pulse power, key sensing indicators can be improved: In 2022, Wu et al. proposed a two-dimensional Wiener filtering image deconvolution scheme, using 40 ns pump pulses on a 1.8 km sensing optical fiber, achieving a spatial resolution of up to 10 cm [55]. A typical BOTDR system is illustrated in Figure 6. In the same year, Liu et al. first applied the sparse representation algorithm based on pre-built dictionaries to BOTDR noise reduction; in a 10.15 km simulation experiment, when adding 5 dBm random noise, the SNR of the non-temperature-changing area increased by 24.3 dB, effectively improving the signal quality and measurement accuracy of BOTDR [56]. In 2023, Wang et al. proposed a spatial adaptive image denoising (SAID) scheme, performing denoising processing on the two-dimensional Brillouin gain spectrum (BGS) distance matrix of BOTDR, under the experimental conditions of a 25.1 km single-mode optical fiber and a 2 m spatial resolution, achieving a 21.92 dB increase in SNR, and significantly reducing the data processing time [57]. 

The detection pulses of the Brillouin optical time domain reflectometer are modulated through the gain switch modulation scheme. In the system, a modulation process based on current-controlled gain state is designed (without the traditional encoding and decoding steps), which can improve the system SNR, reduce the BFS measurement error, and extend the sensing distance without increasing the single-pulse power. In 2019, Bai et al. [58] first applied this gain switch modulation scheme to the BOTDR system. The extinction ratio of the detection pulses reached 51.26 dB, which was 16.11 dB higher than that of the traditional electro-optic modulator (EOM). At a sensing fiber length of 9.941 km, the root mean square error of BFS measurement decreased from 2.49 MHz to 0.78 MHz, and the fluctuation range was reduced from 7.84 MHz to 2.76 MHz. Under the condition of maintaining a spatial resolution of 1 m, the sensing distance of BOTDR was extended from 10.75 km to 27.5 km [58]. 

#### 2.1.3. Rayleigh Optical Time Domain Reflectometry

Distributed sensing based on Rayleigh scattering enables the measurement of events and physical parameters along optical fibers by analyzing intensity, polarization, and phase changes in the backscattered light. Integrated with OTDR technology, this includes intensity-based optical time domain reflectometry (I-OTDR) for locating attenuation, reflections, and breakpoints in fibers; polarization-optical time domain reflectometer (P-OTDR) for measuring temperature, strain, and vibration; and φ-OTDR for detecting vibration and acoustic waves. Currently, in distributed fiber sensing, φ-OTDR has attracted widespread attention for its high sensitivity in vibration and acoustic detection. This review focuses on φ-OTDR due to its dominance in modern vibration sensing applications. However, such systems face two main challenges: first, they suffer from inherent phase noise and external environmental interference, leading to insufficient SNR; second, there exists a fundamental trade-off among spatial resolution, sensing range, and system SNR, and improving spatial resolution typically requires reducing pulse width, which consequently degrades SNR and shortens the sensing distance. To overcome these limitations, researchers are primarily exploring solutions through three directions.

**Figure 7:**
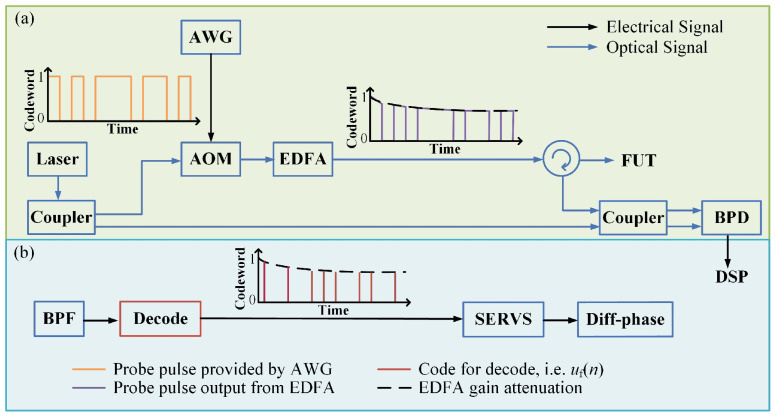
System structure of the GOPC φ-OTDR: (**a**) Schematic diagram of GOPC φ-OTDR structure. (**b**) signal processing flowchart [61].

Encoding modulation converts high-power short pulses into wide-encoded sequences with low peak power for transmission in optical fibers, avoiding nonlinear effects and improving the SNR. At the receiving end, pulse compression technology processes the received encoded sequences, concentrating their energy into an extremely narrow pulse, restoring high spatial resolution, and achieving high resolution and high SNR over long distances. In 2022, Liang et al. [59] applied the optical pulse encoding technology based on mismatch filtering to φ-OTDR. On a 1 km sensing optical fiber, the spatial resolution was restored from the 32 m encoded pulse to 2 m, and the SNR was increased by approximately 6.5 dB [59]. In 2024, Shen et al. [60] applied the sidelobe suppression algorithm based on the least mean square (LMS) adaptive filter to the phase-encoded phase-sensitive optical time domain reflectometer. On a 11 km single-mode optical fiber, a spatial resolution of 3.81 m was achieved, and the peak sidelobe ratio of the signal was improved to −15.86 dB [60]. In the same year, Zhao et al. proposed a genetic-optimized code (Go-code) based optical pulse coding (OPC) φ-OTDR (GOPC φ-OTDR) scheme, achieving a spatial resolution of 10 m over a 10 km sensing distance and improving the phase demodulation SNR by approximately 7.48 dB compared with the traditional Golay encoding system [61]. The diagram depicted in Figure 7 demonstrates the system structure of the GOPC φ-OTDR. The encoded signal is amplified utilizing an EDFA before being transmitted into the fiber under test (FUT). The gain saturation of EDFA results in an attenuation in the intensity of the probe pulse trains, as depicted in Figure 7a. The complete process of Digital Signal Processing (DSP) in GOPC φ-OTDR is carried out as shown in Figure 7b. In 2025, Zuo et al. [62] based on the multi-band chirped pulse (MBCP) scheme, achieved a spatial resolution of 2.4 m on a 50 km sensing distance and significantly improved the SNR dynamic range by 17.1 dB through multi-band and unequal amplitude design, with a strain sensitivity of approximately 0.8 nε/√Hz [62].

By introducing auxiliary reference optical paths or utilizing compensation algorithms, researchers have successfully achieved effective suppression of core noise sources such as laser phase noise, frequency drift, and signal attenuation. These methods demonstrate great potential in improving the SNR of the system, ensuring high spatial resolution, and extending the sensing distance. In 2024, Xiao et al. [63] proposed a signal and core joint phase noise compensation method for the φ-OTDR system based on coherent detection and matched filtering. They achieved a spatial resolution of 4.2 m and a strain resolution of 68 pε/√Hz on a 50 km single-mode optical fiber [63]. In 2025, Ding et al. [64] proposed a spatial-temporal phase compensation (STPC) demodulation method based on Fourier transform method (FTM) for noise suppression. This method replaces traditional orthogonal demodulation and time domain filtering with frequency domain processing, achieving a spatial resolution of less than 1 m within a sensing length range of 2.5 km to 5 km, and suppressing the fading noise to below 1% through adaptive phase compensation [64]. The same year, Jiang et al. proposed a dual-pulse distributed optical fiber sensor based on active laser frequency compensation. They adjusted the laser frequency by monitoring the interference signal of the non-equilibrium Mach–Zehnder interferometer in reverse, compensating for the length jitter of the interference arm and laser frequency drift, reducing the short-term noise of the interference signal by 60% and the noise fluctuation of the demodulated waveform by 50% [65].

The noise reduction algorithm is the core software for enhancing the performance of OTDR. It directly improves the SNR by suppressing random and specific noises in the signal. A higher SNR means that the system can identify more weak effective signals, thereby extending the sensing distance. At the same time, it ensures that the signal remains clear while maintaining high spatial resolution, avoiding the deterioration of the SNR due to reduced pulse energy. In 2021, Bai et al. proposed a fusion noise reduction algorithm based on empirical mode decomposition and time-frequency peak filtering (EMD-TFPF), which increased the positioning SNR of the system under 0.1 Hz low-frequency vibration from 7.6 dB to 37.6 dB and expanded the low-frequency response range of the system to 10–5 Hz [66]. In 2024, Liang et al. [67] proposed a synchronous demodulation and deep learning network for φ-OTDR. It achieved a spatial resolution of 0.9 m at a sensing distance of 10.1 km and reduced the strain noise level to 98 pε/√Hz [67]. In 2025, Tan et al. proposed a multi-step joint noise reduction method, which significantly increased the dynamic range of the SNR by more than 15 dB while maintaining a spatial resolution of 1 m on a 50 m sensing optical fiber [68].

Table 1 presents the performance of OTDR demodulation technology for backscattering-based DFOS networks, detailing the employed method, specific implementation scheme, the achieved metrics, typical benefits, and remaining gaps.

The above OTDR-based demodulation schemes are widely adopted due to their implementation simplicity and strong compatibility with long-range distributed sensing. However, OTDR demodulation is intrinsically constrained by time domain pulse parameters and the range–resolution–SNR trade-off. For applications requiring higher spatial resolution, improved spectral/phase discrimination, or alleviating pulse-width-related constraints, OFDR provides a complementary frequency domain interrogation route. Therefore, the following subsection reviews OFDR-based demodulation advances for Raman, Brillouin, and Rayleigh scattering sensing, using the same problem–solution perspective to facilitate comparison with OTDR counterparts.

### 2.2. OFDR Demodulation Technology

OFDR is a technology that utilizes the interference characteristics of continuous light waves with varying frequencies in the fiber to locate and quantify various event points on the fiber link, as well as the properties of the fiber itself. A continuous laser beam with frequency varying linearly over time is emitted into the fiber, and then the spatial distribution information of the entire fiber is obtained by analyzing the spectrum formed by the interference between the returned scattered light and the reference light. In the system based on OFDR, the spatial resolution is inversely proportional to the frequency scanning range. Increasing the scanning bandwidth can enhance the spatial resolution, but at the same time, it imposes higher requirements on the laser linear tuning stability and coherence length, and the system becomes more sensitive to environmental disturbances. Therefore, the detection distance is usually limited by coherence attenuation and phase noise accumulation.

#### 2.2.1. Raman Optical Frequency Domain Reflectometry

Following the ROTDR sensing discussed in Section 2.1.1, the Raman optical frequency domain reflectometry (ROFDR) provides an alternative frequency domain route that maps modulation frequency to spatial information, enabling different trade-offs in long-distance temperature demodulation. The ROFDR technology, which combines distributed sensing based on Raman scattering with OFDR technology, detects the anti-Stokes light and Stokes light of the backscattered light, respectively, for modulated light of different frequencies. Then, through inverse Fourier transformation, the time domain response information of the system is obtained, and the temperature distribution on the sensing optical fiber is demodulated. The ROFDR technology has higher requirements for frequency modulation and signal processing. Problems such as phase offset and frequency jitter may occur during signal transmission, acquisition, and time–frequency conversion, resulting in a decrease in spatial resolution and demodulation accuracy. Moreover, for long-distance sensing, a wide range of frequency modulation is required, which leads to low signal acquisition and processing efficiency and poor real-time detection performance.

**Figure 8:**
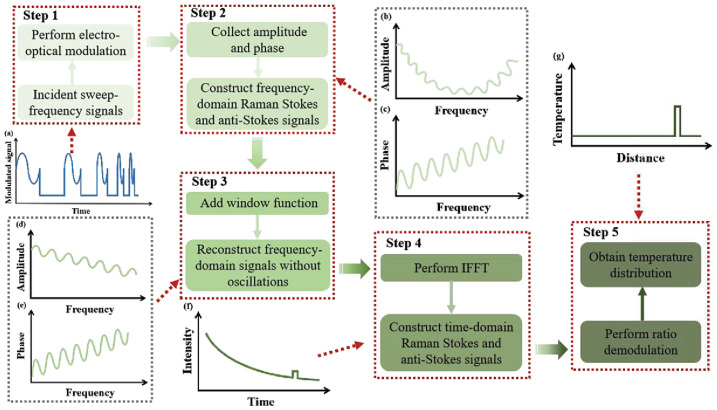
Principle schematic diagram of IROFDR demodulation scheme. (**a**) The electro-optically modulated signal injected into the sensing fiber. (**b**) Amplitude and (**c**) phase of Raman signals in frequency domain. (**d**) Amplitude and (**e**) phase of reconstructed Raman signals in frequency domain. (**f**) Intensity of Raman signals in time domain. (**g**) Distributed temperature demodulation result [70].

By suppressing signal coherence and jitter, the spatial resolution and demodulation accuracy of the ROFDR system in long-distance temperature sensing can be effectively improved. In 2015, Chen et al. proposed to combine the non-coherent ROFDR technology with the three-channel synchronous RF phase-locked amplification technology, achieving a spatial resolution of 0.93 m and a temperature resolution of 0.2 °C on a 2.5 km optical fiber [69]. In 2025, Cao et al. [70] adopted an incoherent Raman optical frequency domain reflection (IROFDR) scheme using a sinusoidal modulated laser with a uniform DC bias step as the detection light. In the time–frequency transformation process, they added a Caesar window function to reconstruct the frequency domain signal to suppress the demodulation deviation caused by signal fluctuations and could achieve a spatial resolution of 0.5 m and a temperature resolution of 0.1 °C at a 10 km sensing distance [70]. As shown in Figure 8, Raman distributed temperature sensing based on the IOFDR scheme is achieved through the implementation of the following five steps. First, the incident light is electro-optically modulated into sweep-frequency light, which is subsequently injected into the sensing fiber to excite Raman backscattering signals, as shown in Figure 8a. Second, as presented in Figure 8b,c, the amplitude and phase of Raman signals are collected, which are utilized to construct the frequency domain Raman Stokes and anti-Stokes signals. Third, the window function was added to reconstruct the Raman signals in the frequency domain without oscillations; the amplitude and phase of the updated Raman signals are expressed in Figure 8d,e. Fourth, an inverse fast Fourier transform (IFFT) brings frequency domain Stokes and anti-Stokes signals to the time domain, as depicted in Figure 8f. Finally, as demonstrated in Figure 8g, distributed temperature is directly calculated from the ratio demodulation of the time domain Raman Stokes and anti-Stokes signals.

By applying compressive sensing technology, the amount of data collected for signal acquisition can be effectively reduced, thereby improving the efficiency of signal processing. In 2024, North et al. [71] first applied compressive sensing technology to ROFDR. When the temperature distribution conformed to the expected model, only less than a quarter of the frequency subsets needed to be collected to complete the reconstruction of temperature distribution information. The experiment verified that this method could still effectively detect temperature changes and distributions over a 4 km long optical fiber, and the matching degree with the full-frequency measurement results was good [71].

#### 2.2.2. Brillouin Optical Frequency Domain Reflectometry

Following the BOTDR sensing discussed in Section 2.1.2, the Brillouin optical frequency domain reflectometry (BOFDR) technology, which combines distributed sensing based on Brillouin scattering with OFDR technology, utilizes the mapping relationship between the frequency domain and the spatial domain to achieve the positioning and measurement of temperature and strain along the optical fiber. Compared with BOTDR technology, BOFDR avoids the limitations of modulation pulse width and phonon lifetime on spatial resolution and sensing distance, circumvents the problem of pulse broadening in the time domain, and has stronger tolerance to interference such as light source fluctuations and fiber bending loss. However, the BOFDR technology relies on vector network analyzers to achieve modulation and detection, which limits its application scenarios and commercialization [72,73].

By simplifying the analog signal processing process through digital signal processing technology, a low-cost solution for BOFDR can be achieved. In 2018, Mohamed et al. proposed a three-filter comb structure and digital envelope detection scheme for the BOFDR technology, significantly reducing the linewidth of the BGS to 32 MHz, which improved the theoretical limit of spatial resolution and stably reconstructed the BFS distribution at a sensing distance of 10 km, while maintaining a spatial resolution of 1 m [74].

#### 2.2.3. Rayleigh Optical Frequency Domain Reflectometry

The combination of distributed sensing based on Rayleigh scattering and OFDR technology can achieve high-precision distributed measurement of parameters such as strain and vibration by utilizing the intensity, phase, and spectral information of the Rayleigh backscattering signal (RBS). Compared with OTDR-based Rayleigh sensing technology discussed in Section 2.1.3, this technology has core advantages such as millimeter-level spatial resolution, high sensitivity, and dynamic range. However, this technology faces performance degradation issues such as frequency sweep nonlinearity, phase noise, and environmental noise, which lead to a decrease in SNR. On the other hand, in terms of key performance indicators such as sensing length, spatial resolution, measurement accuracy, range, and speed, there are often certain constraints and limitations, making it difficult to simultaneously improve all aspects. The performance improvement mainly lies in the following three directions.

**Figure 9:**
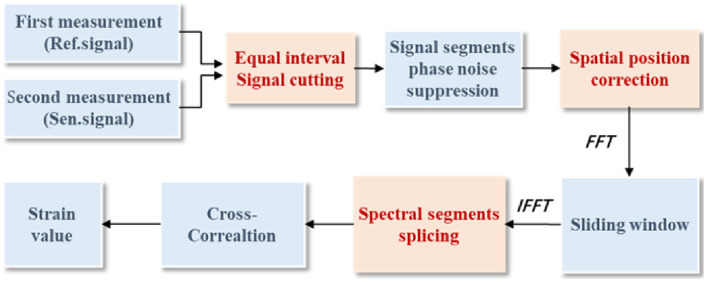
Flow chart of strain demodulation for spectral splicing method [77].

By improving or innovating the demodulation methods and overcoming the constraints among some performance indicators, the application scenarios of OFDR can be expanded. In 2022, Wang et al. proposed the phase accumulation demodulation method, which accumulates the phase changes during the continuous observation of the strain loading process to achieve ultra-large strain demodulation of 14,000 με, with a strain accuracy of 0.48 με [75]. In 2023, Zhang et al. proposed the polarization diversity phase demodulation technology, which synchronously collects RBS signals through dual polarization channels to suppress the signal distortion caused by polarization fading, achieving a strain accuracy of ±1.1 με and a measurement range of 400 με [76]. In 2023, Zhu et al. [77] proposed the spectral splicing method, which splits the wide-spectrum swept-frequency signal into multiple narrow-spectrum segments, compensates for phase noise section by section, and then stitches to restore the complete spectrum. At a sensing distance of 1170 m, it achieves a spatial resolution of 1 cm and a strain accuracy of ±3.2 με, with a strain range of 10,000 με [77]. The specific process of the spectral splicing method is shown in Figure 9. First, the first and second measurements were performed before and after the application of strain to obtain two-time domain Rayleigh scattering signals, reference signal and sensor signal, respectively. Second, an equally spaced cut of reference and sensor signal. Third, phase noise correction is performed separately for each signal segment after the reference and sensor signal cuts to eliminate phase noise in each segment. Fourth, calculate the correction coefficients and correct the spatial position of each signal segment of the reference and sensor signal, respectively. Fifth, a fast Fourier transform transforms each signal segment of the reference and sensor signal from the time domain to the spatial domain. And sliding intercepts according to a sliding window, where the width of the sliding window indicates the sensed spatial resolution. Sixth, the signals intercepted on each signal segment of reference and sensor signal is converted from the spatial domain to the spectral domain by inverse Fourier. And spectral splicing is performed separately. Seventh, strain values are obtained by cross-correlation according to splicing spectra [77]. In 2023, Lin et al. [78] proposed to combine the spectral cursor technology with OFDR technology, using a broadband master scale and narrowband cursor modulation mode to overcome the constraints of frequency response and amplitude range. At a sensing distance of 10 m, it achieves a large strain measurement of 10,856 με, with a frequency response of 1.21 kHz and a sensitivity of 18.7 nε/√Hz [78].

To address issues such as the non-linearity of frequency sweeping caused by the defect of the light source scanning mechanism and the broadening of the spatial point spread function due to the inherent phase noise of the light source and environmental interference, researchers have achieved noise suppression and frequency sweeping non-linearity correction through hardware calibration or algorithm compensation. They have optimized the spectral quality of the RBS during modulation and transmission, thereby improving the sensing performance. In 2019, Xing et al. [79] proposed a time-scale factor correction method, which effectively compensates for the non-periodic frequency sweeping non-linearity by extracting the light frequency variation pattern from the auxiliary interferometer signal. At a sensing distance of 155 m, the spatial resolution was optimized to 0.17 mm, and the measurement capacity reached 9.12 × 10^5^ [79]. In 2022, Zou et al. [80] combined high-precision periodic phase noise estimation (PPNE) with de-slope filtering and precisely fitted the light source phase noise through the third-order Taylor expansion to suppress the random noise amplification effect in the high-order differentiation process. At a sensing distance of 8 km, while maintaining a sub-millimeter spatial resolution, the measurement capacity exceeded 107, solving the problem of resolution drop caused by the accumulation of phase noise at long distances [80].

The researchers also conducted extensive work on the noise reduction algorithms in the demodulation process, optimizing the quality of RBS signals through digital signal processing, suppressing noise interference, and improving sensing performance. In 2019, Feng et al. applied the wavelet noise reduction algorithm to OFDR signal processing, reducing the root mean square error of strain measurement from 8.7 με to 2.3 με on a 300 m optical fiber [81]. In 2020, Zhao et al. first applied the three-dimensional image noise reduction idea to OFDR, removing noise points in the RBS three-dimensional image using the total variation method and improving the strain demodulation accuracy by 30% on a 50 m optical fiber [82]. In 2022, Pan et al. processed the redundant information of three-dimensional images through block matching and principal component analysis, increasing the strain accuracy from 1.52 με to 0.31 με at a spatial resolution of 5 cm and increasing the accuracy by approximately five times [83]. In 2022, Feng et al. proposed the Kalman prediction algorithm, combining local search to optimize the demodulation process, achieving a temperature accuracy of 0.5 °C and a strain accuracy of ±5 με on a 50 m optical fiber, and increasing the data processing rate by 50% [84].

Table 2 assesses the performance of OFDR demodulation technology for backscattering-based DFOS networks, detailing the employed method, specific implementation scheme, the achieved metrics, typical benefits, and remaining gaps.

Overall, the reviewed studies on backscattering-based DFOS indicate that the evolution of demodulation technologies is increasingly characterized by system-level trade-off management rather than isolated performance enhancement. For OTDR-based schemes, including Rayleigh-, Brillouin-, and phase-sensitive implementations, demodulation performance is often constrained by weak backscattered signals; cumulative noise effects over long distances; and the intrinsic tension among sensing range, spatial resolution, and demodulation accuracy. As a result, recent efforts have focused on coordinated improvements in pulse design, coding strategies, coherent detection, noise suppression, and data reconstruction algorithms, with the goal of improving measurement reliability under long-range and dynamic operating conditions. In contrast, OFDR-based backscattering systems exhibit clear advantages in ultra-high spatial resolution and fine spectral/phase discrimination, but their practical demodulation performance is more strongly influenced by sweep nonlinearity, phase instability, large data volume, and computational complexity, especially in real-time or large-scale scenarios. Consequently, many recent studies have shifted from purely optical optimization to joint optical–digital approaches, where calibration, compensation, and fast signal processing are integrated into the demodulation chain. Taken together, these developments suggest a common trend in backscattering-based DFOS: demodulation is no longer a downstream signal interpretation step but a central design dimension that determines how effectively the sensing system can approach its theoretical performance limits under realistic deployment constraints. Meanwhile, in response to the difficulty of achieving high-precision multi-parameter measurements with a single scattering mechanism, multi-system integration has emerged as an important direction. By combining the distinct physical sensitivities of Rayleigh, Brillouin, and Raman scattering mechanisms, it enables decoupled monitoring and coordinated sensing of parameters such as temperature, strain, and vibration [85,86,87].

## 3. UWFBG-Based DFOS Networks

UWFBG networks have emerged as a powerful solution to bridge the gap between discrete point sensors and fully distributed systems. Unlike traditional FBG sensors, UWFBG arrays feature ultra-low reflectivity (typically below −40 dB) and high integration density (spacing from centimeters to meters), effectively constructing a continuous sensing channel similar to backscattering fibers but with significantly enhanced signal strength. Unlike intrinsic scattering, which relies on different physical mechanisms (Raman/Brillouin/Rayleigh) for different parameters, UWFBG networks primarily rely on the interrogation method (φ-OTDR, OTDR, and OFDR) to determine the sensing capabilities.

The first is fully distributed UWFBG sensing based on φ-OTDR. By utilizing the coherent interference of light reflected from the grating array, this approach treats the UWFBG as stable, high-reflectivity discrete reflection. It enables high-fidelity distributed acoustic and vibration sensing, overcoming the fundamental limits of low SNR and signal fading inherent to intrinsic Rayleigh scattering.

The second is quasi-distributed UWFBG sensing based on wavelength demodulation. This method utilizes the center wavelength shift of individual gratings to measure static parameters such as temperature and strain. Although physically discrete, the massive multiplexing capacity of UWFBG arrays allows for high-density coverage that approximates distributed sensing. However, its essence remains quasi-distributed sensing, enabling precise measurement of high-density points. For this category, two primary interrogation approaches are employed: OTDR and OFDR.

This section follows a consistent problem–solution narrative for UWFBG-based DFOS demodulation. For each interrogation type (φ-OTDR-, OTDR-, and OFDR-based UWFBG systems), we first summarize the fundamental limitations and coupled trade-offs that dominate practical performance, such as channel discrimination under multi-point reflections, multiplexing capacity, phase/wavelength tracking stability, and the range–resolution–speed constraints in dense sensing networks. We then group representative advances into recurring solution families, including (i) interrogation architecture and hardware-level modulation/compensation; (ii) multiplexing and coding strategies to improve efficiency and suppress crosstalk; and (iii) algorithmic processing for denoising, reconstruction, and robustness enhancement. In addition, subsection-level summaries are strengthened by revised tables that highlight not only performance metrics but also key advances and remaining gaps, enabling readers to quickly extract trends and identify open challenges across different UWFBG demodulation routes.

### 3.1. φ-OTDR Demodulation Technology

Traditional φ-OTDR systems rely on intrinsic Rayleigh backscattering, which suffers from extremely weak signal intensity (~−80 dB/m) and random polarization fading, limiting the sensing distance and signal reconstruction quality. Incorporating UWFBG arrays into φ-OTDR systems fundamentally transforms the sensing mechanism from random scattering to discrete reflection.

Current φ-OTDR schemes are primarily classified into heterodyne detection and direct detection (homodyne detection) [88]. In heterodyne detection, a local oscillator (LO) light interferes with the backscattered signal shifted by a frequency ∆f, allowing for phase extraction via I/Q demodulation or Hilbert transform. Conversely, direct detection schemes typically rely on double-pulse interference without an LO. Despite the theoretical advantages, achieving high SNR and large dynamic range remains challenging. The system performance is often constrained by the frequency noise of the laser source, which manifests as a non-negligible noise floor, and by the phase wrapping errors that limit the measurable dynamic range of vibration amplitudes.

#### 3.1.1. Signal-to-Noise Ratio Enhancement

SNR is the cornerstone of high-fidelity reconstruction in UWFBG-based φ-OTDR systems. Enhancing SNR primarily involves suppressing system noise floors (such as laser phase noise) and mitigating signal fluctuations caused by environmental or polarization instabilities.

Phase Noise Compensation: Laser frequency drift is a dominant noise source in phase demodulation, often burying low-frequency strain signals. In 2018, Wu et al. [89] pioneered a phase noise compensation structure utilizing an auxiliary interferometer within a UWFBG sensing network. By correlating and subtracting the source phase jitter, they achieved an equivalent phase noise floor as low as −71.2 dB re rad/√Hz over a 20 km sensing distance, significantly enhancing the system’s low-frequency detection limit.

Advanced Modulation and Coding: Pulse modulation schemes have been pivotal in boosting effective signal strength. In 2021, Tang et al. [90] proposed a dual-pulse detection scheme to suppress crosstalk, improving the SNR to 45.92 dB at 0.1 Hz and 43.33 dB at 30 kHz. Building on this, Tang et al. [91] in 2022 introduced a Golay coding modulation technique for adjacent grating phase interferometry. By modulating the probe pulse sequences, they successfully enhanced the vibration signal SNR to 62.2 dB at 100 Hz, demonstrating the efficacy of coding gain in discrete reflector arrays.

**Figure 10:**
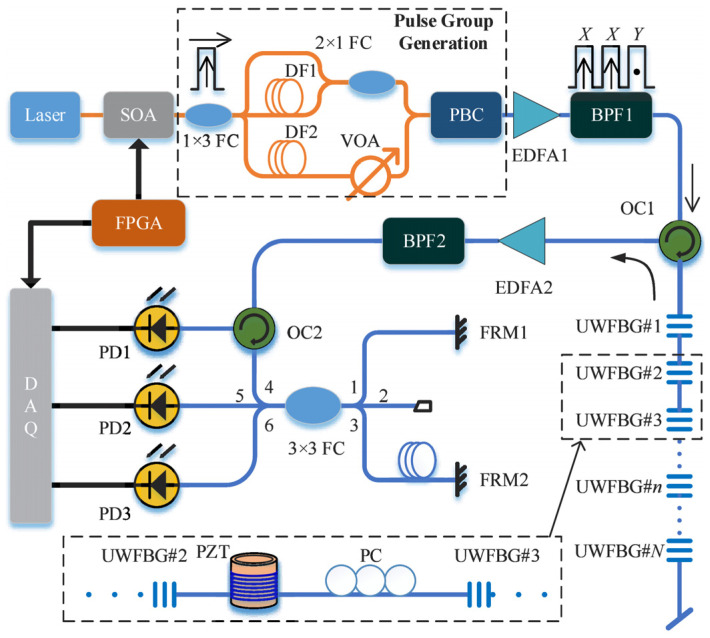
The experimental setup for distributed interferometric sensing with polarization-fading suppression. SOA: semiconductor optical amplifier; FC: fiber coupler; DF: delay fiber; VOA: variable optical attenuator; PBC: polarization beam combiner; EDFA: erbium-doped fiber amplifier; BPF: bandpass filter; OC: optical circulator; PD: photodetectors; FPGA: field-programmable gate array; DAQ: data acquisition card; PZT: piezoelectric transducer; PC: polarization controller [92].

Polarization Fading Suppression: Polarization mismatch can lead to severe signal fluctuations. Fu et al. [92] in 2022 developed a polarization diversity scheme that interrogates the array with orthogonal polarization pulses. By dynamically selecting the channel with higher visibility, they maintained a stable SNR exceeding 30 dB over long-term operation, effectively eliminating polarization-induced blind spots. The experimental setup in Figure 10 is adopted to investigate the proposed polarization fading suppression method.

#### 3.1.2. Dynamic Range Optimization

The dynamic range of φ-OTDR systems is fundamentally constrained by the phase wrapping ambiguity and the sampling rate of the system. When the vibration amplitude induces a phase shift exceeding these limits, standard demodulation algorithms fail, leading to signal distortion. Consequently, extending the linear measurement range without compromising sensitivity has become a key research focus.

Multi-Wavelength and Optical Unwrapping: Overcoming the intrinsic phase limit often requires hardware-assisted strategies. In 2019, Fu et al. [93] achieved a significant breakthrough in large dynamic range vibration sensing by employing a dual-wavelength probing scheme. By performing linear regression on the phase differences obtained from two distinct wavelengths, they successfully unwrapped the phase beyond the conventional π-constraint, enabling the faithful reconstruction of large-amplitude vibrations. Similarly, chirped-pulse interrogation schemes [94] have been utilized to map frequency shifts to time delays, thereby expanding the measurable range of dynamic strains while maintaining high spatial resolution.

Algorithmic Phase Unwrapping: Advanced digital signal processing provides a software-defined path to dynamic range extension. The differential-unwrapping-integral algorithm [95] has proven effective in mitigating projection errors during high-slew-rate events, robustly recovering signals that would otherwise suffer from wrapping artifacts. Guo et al. innovatively propose a least squares linear programming phase unwrapping (ls-LPPU) algorithm, which determines the wrapping orders under large dynamic strains by minimizing the squared optical path difference between two wavelengths, thereby achieving temporally independent phase unwrapping. The proposed method achieves precise restoration of large dynamic strains ranging from 13.8 to 44.6 με, representing an enhancement of at least 90-fold. Its temporally independent characteristic eliminates the π-phase constraint, achieving the decoupling of dynamic strain extension and frequency response bandwidth [96].

Deep Learning-Enhanced Unwrapping: More recently, data-driven approaches have been introduced to address nonlinearities in phase demodulation. Peng et al. proposed a deep neural network-based 2-D phase unwrapping method, demonstrating the ability to predict and correct wrapping errors in real-time, offering a promising direction for achieving ultra-large dynamic range in complex noise environments [97].

Table 3 presents an overview of the recently developed φ-OTDR demodulation technology for UWFBG-based DFOS networks, detailing the optimization direction, methodology, specific implementation scheme, key performance advances, and remaining gaps.

Based on the above φ-OTDR-based UWFBG demodulation advances, this route is particularly effective for dynamic event monitoring due to its high phase sensitivity and strong temporal response. Nevertheless, φ-OTDR-based schemes often face practical constraints related to phase stability, unwrapping reliability, and robustness under long-term field drift, especially when the sensing network scales to dense grating arrays. In many quasi-distributed deployment scenarios, the dominant requirement shifts from maximizing dynamic response to improving multiplexing efficiency, channel discrimination, and stable readout across a large number of sensing points. Therefore, the following subsection reviews OTDR-based UWFBG interrogation and demodulation methods, which emphasize scalable multiplexing architectures and efficient reconstruction for large-scale quasi-distributed sensing networks.

### 3.2. OTDR Demodulation Technology

OTDR technology is the most commonly used demodulation method for quasi-distributed grating wavelength information networking. This method distinguishes the serially connected grating array within the time domain by leveraging the temporal delays of reflected light originating from gratings situated at varying spatial locations. Following this temporal separation, the complete reflection spectrum of the grating array is reconstructed by integrating the intensities of reflected pulses corresponding to optical pulses of distinct wavelengths, thereby enabling the localization and demodulation of sensing information.

The online fabrication technology of grating array fibers enables the production of gratings with ultra-weak reflectivity, which reduces optical power attenuation and inter-grating crosstalk. The combination of OTDR technology and the online fabrication of grating array fibers significantly enhances the multiplexing capacity of quasi-distributed wavelength demodulation systems for these fibers. In 2012, Wang et al. [98] proposed a time-division multiplexing sensing network utilizing UWFBGs, achieving multiplexing of over one thousand sensors on a single optical fiber.

Nevertheless, the spatial resolution of conventional OTDR systems is limited by the pulse width of the probing optical pulses, while the demodulation speed is constrained by the laser’s wavelength tuning rate and the signal processing speed.

#### 3.2.1. Spatial Resolution Optimization

To overcome the spatial resolution limitation, researchers have primarily explored two main approaches.

**Figure 11:**
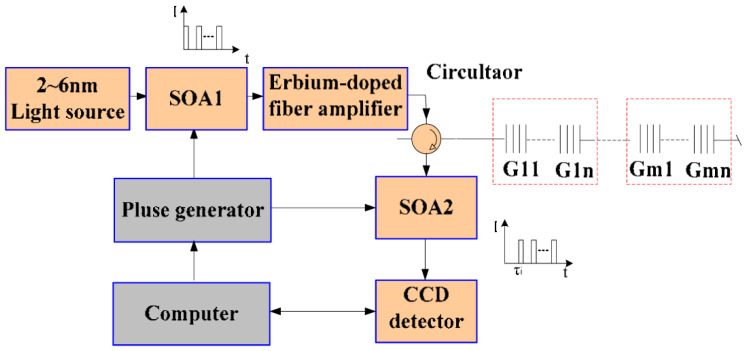
Sensor network with an ultra-weak TDM + WDM-FBG array [100].

The primary methods for enhancing spatial resolution include hybrid multiplexing technology and aliased spectrum demodulation technology. By integrating techniques such as time division multiplexing (TDM), wavelength division multiplexing (WDM), and space division multiplexing (SDM), spectral overlap between adjacent gratings is prevented, thereby overcoming the limitations imposed by a single time domain. In 2012, Dai et al. [99] integrated semiconductor optical amplifier (SOA) resonant cavity technology to realize the demodulation of a TDM–WDM FBGs sensor network. The sensor network was composed of multiple groups of FBGs, employing TDM for inter-group access and WDM for intra-group access. The system achieved a spatial resolution of 0.2 m, facilitating the multiplexing of up to 1000 sensors on a single optical fiber. This advancement significantly enhanced both the multiplexing capacity and spatial resolution of the sensing network. In 2013, Luo et al. [100] proposed a high-speed imaging spectral demodulation method, which realized the demodulation of a WDM–TDM hybrid multiplexing sensing network using an InGaAs linear image sensor manufactured by Bayspec (USA) as the core component. As shown in Figure 11, it is a sensor network with an ultra-weak TDM + WDM-FBG array. This system achieved a spatial resolution of 2 m and a response speed of 5000 FBGs demodulated per second [100,101]. In 2018, Yang et al. [102] reported a grating array fiber temperature sensing network based on TDM–WDM networking, achieving a spatial resolution of 1 m within a detection range of 2 km.

Aliased spectrum demodulation technology allows grating spectra to be aliased in the time domain, with wavelength information subsequently extracted through algorithmic processing. In 2020, Wang et al. [103] proposed an OTDR demodulation strategy with relaxed pulse width based on a UWFBG array. Combined with a fiber grating segmented spectrum reconstruction method, this strategy achieved a spatial sensing resolution of 10 cm and a positioning resolution of 1 m under a 10 ns probe pulse, with a detection distance of 2 km. In 2023, Jiang et al. [104] proposed a rapid wavelength detection method combining a one-dimensional deep convolutional neural network. By integrating OTDR reflected signals with a neural network spectrum reconstruction model, high-precision wavelength demodulation of serially overlapping spectra was achieved, with a system spatial resolution of 10 cm.

#### 3.2.2. Demodulation Speed Enhancement

To enhance the demodulation speed of OTDR quasi-distributed gratings, the key approach is to optimize the efficiency of converting grating wavelength information into time domain information. Certain researchers employ a demodulation approach utilizing dispersion compensating fiber (DCF). The fundamental principle underlying this method involves transforming the wavelength variation of the fiber Bragg grating into a corresponding change in delay time. Subsequently, the system performs wavelength demodulation for each fiber Bragg grating by analyzing the magnitude of the induced delay. In 2015, Li et al. [105] employed a pulsed laser source and exploited the dispersion delay effect of DCF to perform wavelength-to-time conversion. By transmitting a single optical pulse, they simultaneously acquire wavelength information from a quasi-distributed grating array, resulting in a system demodulation error of 27.8 pm and a demodulation rate of 100 kHz. In 2016, Ma et al. [106] implemented high-speed demodulation of FBG arrays utilizing the pulse dispersion technique, achieving rapid demodulation of large-capacity identical FBG arrays at a frequency of 20 kHz.

Furthermore, a demodulation approach utilizing an edge filter can be implemented. This technique converts the wavelength variations of FBGs into temporal intensity fluctuations. In 2022, Ding et al. [107] proposed a method that integrates pulsed light sources, edge filtering, and OTDR technology. The approach employed a high birefringence fiber loop mirror (HiBi-FLM) configuration to assign a distinct edge filter to each WDM-FBG. Consequently, the reflection spectrum of each FBG was translated into corresponding light intensity variations, enabling quasi-distributed grating wavelength demodulation. The system demonstrated a demodulation rate of up to 200 kHz.

Another method involves increasing the wavelength scanning speed of the swept laser, thereby reducing the time domain bandwidth associated with each grating wavelength. This approach enables the acquisition of a complete time-division spectrum of the grating array within a single scan, obviating the necessity for multiple polling scans and the subsequent averaging and fitting calculations. In 2018, Liu et al. [108] advanced the field by developing a Fourier domain mode locked (FDML) high-speed wavelength-swept laser, overcoming the speed limitations inherent to short-cavity swept lasers. By implementing TDM/WDM encoding of the FBG array, the ambiguity between grating position and wavelength information was eliminated. This approach ensured that the demodulation frequency of the WDM/TDM FBG array depended solely on the sweep frequency of the laser. Consequently, high-speed demodulation of multi-parameter physical information, including temperature and vibration, from the FBG array was achieved at 100 kHz. 

Table 4 presents an overview of the recently developed OTDR demodulation technology for UWFBG-based DFOS networks, detailing the methodology, specific implementation scheme, the achieved metrics, typical benefits, and remaining gaps.

Based on the above OTDR-based UWFBG interrogation schemes, significant progress has been achieved in multiplexing capacity, scanning efficiency, and quasi-distributed deployment. Nevertheless, OTDR-based demodulation is still fundamentally constrained by time domain pulse parameters and the associated range–resolution–speed trade-off, and its wavelength/strain/temperature extraction is often limited by intensity fluctuations, crosstalk, and long-term stability in dense grating arrays. For applications demanding higher demodulation accuracy, finer spatial discrimination, and more stable wavelength tracking under high sensor density, OFDR provides a complementary frequency domain interrogation route. Therefore, the following subsection reviews UWFBG-based OFDR demodulation technologies, focusing on how spectral domain reconstruction and fast tracking strategies reshape the accuracy–speed–complexity trade-off compared with OTDR counterparts.

### 3.3. OFDR Demodulation Technology

The utilization of OFDR technology for the demodulation of grating arrays was initially introduced by Froggatt et al. [109] in 1996. This approach fundamentally relies on retrieving the grating impulse response through frequency domain analysis. The system employs a linearly swept laser source; by detecting the beat frequency signal generated by the interference between light reflected from the sensing arm and the reference arm, it is possible to spatially resolve gratings at distinct locations based on their frequency components. Furthermore, the wavelength information is demodulated by analyzing the amplitude and phase of the detected signal. Due to its interference-based operation, OFDR allows the overlap of gratings in both temporal and spectral domains, thereby achieving exceptionally high spatial resolution on the order of sub-millimeters. In a typical OFDR demodulation system, a linearly swept laser emits light with a frequency that varies linearly over time. This swept light is incident into the FBG sensing array and a Faraday rotating mirror (FRM) via a coupler. The light reflected from these two paths undergoes beat frequency interference at the coupler. Because the laser’s emitted light frequency changes linearly over time, individual gratings can be identified by detecting the frequency of the resulting beat signal. Moreover, the wavelengths of the FBGs can be demodulated by analyzing the amplitude and phase characteristics of the beat frequency signal. Due to the interference principle employed in the OFDR demodulation system, FBGs can overlap in both the time and wavelength domains, enabling the system to achieve extremely high spatial resolution, even reaching sub-millimeter levels.

However, the demodulation accuracy of OFDR systems strongly depends on the linearity of laser sweeping, while the demodulation speed is constrained by the laser scanning time and the computational demands of complex backend signal processing algorithms (e.g., cross-correlation calculation and Fourier transform). In recent years, breakthroughs in OFDR technology have primarily focused on the following two aspects.

#### 3.3.1. Demodulation Accuracy Optimization

The key to improving the demodulation accuracy of the OFDR system lies in optimizing laser performance and compensating for sweep frequency nonlinearity. In 2024, Chen et al. [110] proposed a method combining OFDR technology with an FBG array. By compensating for the nonlinear scanning of the laser source through an auxiliary optical path, they achieved distributed strain measurement with high spatial resolution and a large measurement range, attaining a spatial resolution of 1.28 mm. In 2025, Sun et al. [111] employed a tunable laser source with a wavelength resolution of 0.1 pm, compensated for the sweep nonlinearity of the light source using an auxiliary interferometer, and leveraged the ultra-narrowband spectral characteristics of phase-shifted FBGs. This approach significantly improved the accuracy of the distributed temperature measurement to 0.06 °C, with a single-point measurement accuracy of 0.01 °C, effectively overcoming the high-precision demodulation bottleneck of OFDR technology applied to grating array fibers. 

#### 3.3.2. Demodulation Speed Enhancement

The primary approaches to enhancing OFDR demodulation speed include signal processing algorithm optimization and hardware acceleration. In 2018, Zhu et al. [112] proposed a fast demodulation method based on weighted sliding window Fourier transform and logical activation function threshold processing for an OFDR sensing system based on long FBGs. While maintaining a spatial resolution of 0.3 mm, this method increased the demodulation speed to 24 times that of the traditional short-time Fourier transform method and simultaneously achieved a wavelength demodulation accuracy of 11.6 pm, realizing a good balance between high spatial resolution, high precision, and real-time performance.

**Figure 12:**
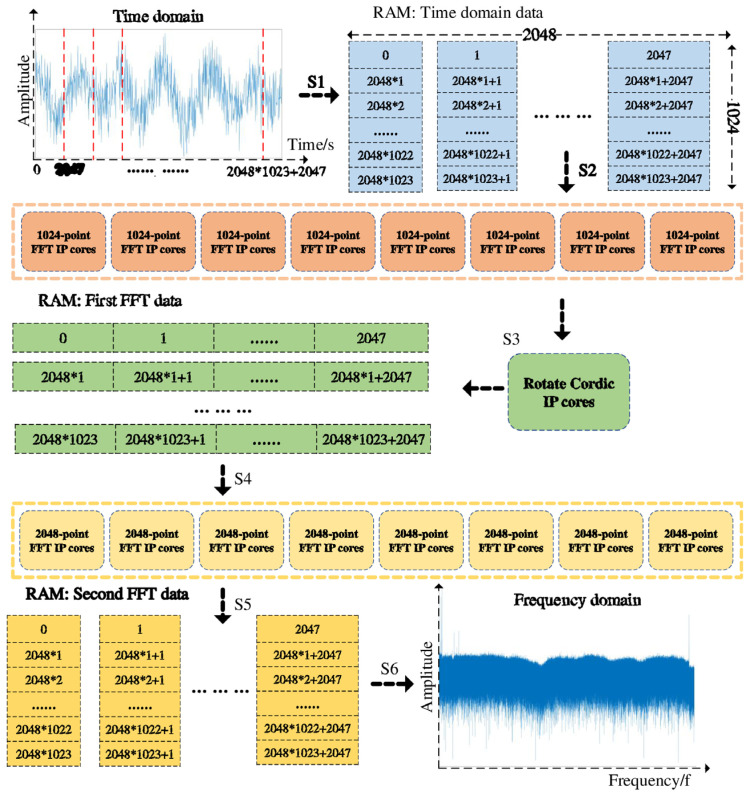
Schematic diagram of 2D FFT algorithm [113].

In 2024, Wang et al. [113] proposed an FPGA-based two-dimensional FFT and frequency domain cross-correlation algorithm. By optimizing the utilization of hardware resources, real-time strain demodulation at 24 Hz was achieved within a sensing length of 50 m and a spatial resolution of 6.4 mm. This solved the problem of insufficient real-time performance in long-distance, high-resolution OFDR systems, providing a feasible real-time sensing solution for health monitoring of large structures such as array antennas. The implementation of the 2M-point 2D FFT algorithm through FPGA is mainly divided into six steps, and the schematic diagram is shown in Figure 12. Step 1, Convert the 2M-point one-dimensional time-domain data into a matrix of 1024 rows and 2048 columns, and store these in the RAM. Step 2, Read each of the 2048 columns of data from the matrix. Configure eight 1024-point FFT IP cores, and perform FFT calculations in parallel. Step 3, Configure the Cordic IP core to multiply the data points computed by the one-dimensional FFT with the corresponding rotation factors in complex numbers. The calculation results are stored in RAM on a column-by-column basis. Step 4, Read the results of the 1024 rows of the matrix by row, configure eight 2048-point FFT IP cores, and perform FFT calculations in parallel. Step 5, The calculation results are stored in the RAM on a row-by-row basis. Step 6, After the second FFT is calculated for all the rows and saved, the data are read out by column to obtain the 2M-point data FFT calculation results [113]. In the same year, Chen et al. [110] used UWFBGs with a spacing of 1 mm as sensing units, combined with an OFDR system, and replaced the traditional cross-correlation operation with a direct spectral peak-finding algorithm. This increased the FBG demodulation speed to 38 Hz, solved the engineering bottleneck of OFDR system demodulation speed, realized real-time capture of dynamic strain processes, and significantly enhanced the feasibility of OFDR combined with FBG technology in engineering applications.

Table 5 presents an overview of the recently developed OFDR demodulation technology for UWFBG-based DFOS networks, detailing the optimization direction, methodology, the achieved metrics, typical benefits, and remaining gaps.

Compared with intrinsic backscattering-based DFOS, the demodulation development path of UWFBG-based DFOS networks reflects a different optimization emphasis. Owing to the discrete and enhanced reflection characteristics of ultra-weak FBG arrays, the signal observables are generally more controllable, which can improve measurement sensitivity and reduce some limitations associated with random backscattering fluctuations. However, this advantage also shifts the core technical challenges toward interrogation architecture design, multiplexing capacity, wavelength/phase interrogation efficiency, and high-speed reconstruction for dense sensing networks. In particular, φ-OTDR-based UWFBG systems are mainly developed for dynamic event monitoring, where demodulation strategies focus on improving SNR, phase stability, and dynamic response capability; OTDR-based UWFBG systems are more closely related to large-scale quasi-distributed deployment, where the key issues include scanning efficiency, channel discrimination, and robust demodulation under multi-point reflection conditions; and OFDR-based UWFBG systems mainly exploit high-resolution spectral interrogation, where the demodulation bottlenecks are associated with accurate peak/wavelength tracking, spectral overlap management, and fast processing for dense grating arrays. Therefore, the value of demodulation advances in UWFBG-based DFOS lies not only in improving absolute measurement accuracy but also in enabling higher sensor density, faster response, and more application-oriented network configurations. This also indicates that future UWFBG demodulation research will likely continue to evolve toward integrated optimization of multiplexing strategy, interrogation hardware, and intelligent signal processing.

## 4. Conclusions

This review has summarized recent advances in demodulation technologies for DFOS networks, covering both backscattering-based and UWFBG-based approaches. Beyond the classification of sensing principles and interrogation architectures, the reviewed literature reveals a common evolution trend: the performance improvement of DFOS systems is increasingly determined by how effectively demodulation strategies coordinate multiple coupled constraints, including SNR, spatial resolution, sensing range, demodulation speed, and stability under practical operating conditions. In this context, demodulation should be regarded not only as a signal interpretation stage but also as a core system-level design dimension that significantly influences the achievable performance boundary of DFOS networks.

From the perspective of system deployment, backscattering-based and UWFBG-based DFOS represent two complementary technical routes with different optimization priorities. Backscattering-based systems provide important advantages in fully distributed coverage and long-range monitoring, but their demodulation performance is often strongly affected by weak signal intensity; noise accumulation; and the trade-offs between sensing distance, temporal response, and accuracy. By contrast, UWFBG-based systems benefit from improved signal controllability and flexible multiplexing configurations, while placing higher demands on interrogation architecture design, channel discrimination, and efficient phase/wavelength reconstruction in dense sensing networks. Despite these differences, a shared development trend can be identified in both routes: demodulation performance increasingly depends on the coordinated optimization of optical architecture, signal acquisition, compensation strategies, and digital/intelligent processing, rather than on isolated improvements in a single subsystem.

Based on the reviewed progress, several directions are expected to be important for the next stage of DFOS development. First, application-oriented co-design of sensing architecture and demodulation algorithms will become increasingly necessary for meeting diverse requirements in smart infrastructure monitoring. Second, more standardized benchmarking protocols and more consistent reporting of key metrics are needed to support fair evaluation across different demodulation schemes. Third, robust real-time demodulation supported by edge computing and hardware acceleration is likely to play a critical role in large-scale engineering deployment. Finally, intelligent demodulation methods are expected to continue developing toward better generalization, interpretability, and stability under changing field environments. These directions will help translate recent laboratory advances into scalable, reliable, and application-ready DFOS networks.

## Figures and Tables

**Table 1:** The performance of OTDR demodulation technology for backscattering-based DFOS networks.

	Method	Concrete Scheme	Accuracy/ Sensitivity	Spatial Resolution	Length	Typical Benefits	Remaining Gaps
ROTDR	Pulse coding	Quasi-periodic pulse encoding [38]	3 °C	1 m	26 km	SNR gain, longer reach	Decode load, rate drop
		Cyclic pseudo-random pulse sequence encoding [39]	1.5 °C	2 m	\		
		Genetic-optimized aperiodic code [35]	0.5 °C	\	40 km		
		Enhanced anti-distortion coding [27]	0.91 °C	1.58 m	70 km		
	Compressed correlation demodulation	ASE + compression correlation demodulation [41]	\	7.5 mm	\	Breaks pulse-width-limited resolution	Computing time consumption
		Chaotic laser + compression correlation demodulation [42]	0.1 °C	5 mm	\		
		Chaos correlation demodulation [43]	\	0.3 m	\		
		Chaos double-correlation demodulation [44]	\	0.1 m	1.4 km		
	Digital signal processing	Improved wavelet transform modulus maxima denoising and dynamic difference denoising method [46]	1.58 °C	\	10.4 km	Better estimation stability	Robustness across conditions
		1DDCNN [47]	0.7 °C	3 m	10 km		
		Fast-NLM [48]	0.42 °C	\	9.68 km		
BOTDR	Pulse coding	Golay complementary coding + FFT [49]		2 m	10 km	Improves long-range SNR	Decoding complexity
		Differential cross-spectrum technique [50]	3.2 MHz BFS	0.2 m	350 m		
		Golay coding + differential cross-spectrum [51]	3.47 MHz BFS	0.4 m	1280 m		
		Random encoding scheme [52]	\	2 m	64.76 km		
		The differential cross-spectrum technique [53]	5.3 MHz BFS	0.4 m	1.2 km		
		Simplex encoding + STFT [54]	1.34 MHz BFS	4.57 m	80.3 km		
	Post-processing technology	Two-dimensional Wiener filtering image deconvolution [55]	\	0.1 m	1.8 km	Noise suppression, cleaner profiles	Potential detail loss/bias
		Spatially adaptive image denoising [57]	\	2 m	25.1 km		
φ-OTDR	Pulse code	Optical pulse encoding technology based on mismatch filtering [59]	\	2 m	1 km	Improves long-range SNR	Decoding complexity
		Sidelobe suppression algorithm [60]	\	3.81 m	11 km		
		Genetic optimization encoding [61]	\	10 m	10 km		
		Multi-band chirped pulse [62]	0.8 nε/√Hz.	2.4 m	50 km		
	Auxiliary reference optical path	Signal-and-kernel phase noise compensation method [63]	68 pε/√Hz	4.2 m	50 km	Phase stabilization, lower drift	Systematic complexity
	Noise reduction algorithm	EMD-TFPF [66]	\	\	10 km	SNR boost, weak-event detect	Generalization, training data requirements
		Synchronous demodulation and noise reduction deep learning network [67]	98 pε/√Hz	0.9 m	10.1 km		
		Multi-step joint noise reduction [68]	\	1 m	50 m		

**Table 2:** The performance of OFDR demodulation technology for backscattering-based DFOS networks.

	Method	Concrete Scheme	Accuracy/ Sensitivity	Spatial Resolution	Length	Typical Benefits	Remaining Gaps
ROFDR	Suppressing signal coherence and jitter	Non-coherent ROFDR + three-channel synchronous RF phase-locked amplification technology [69]	0.2 °C	0.93 m	2.5 km	Mitigates coherent fading/jitter	Added complexity; long-term drift still needs control
		Non-coherent ROFDR + sinusoidal modulated laser [70]	0.1 °C	0.5 m	10 km		
	Compressive sensing technology	ROFDR + compressive sensing technology [71]	\	\	4 km	Reduces sampling burden	Noise sensitivity
BOFDR	Digital signal processing	Three-filter comb structure + digital envelope detection scheme [74]	32 MHz BFS	1 m	10 km	Enhances robustness to disturbances	Computation-heavy for high resolution
OFDR	Improving or innovating the demodulation methods	Phase accumulation demodulation [75]	14,000 με/ 0.48 με	\	\	Expands usable operating range	Complex processing chain; error propagation and calibration
		Polarization diversity phase demodulation [76]	400 με/ ± 1.1 με	\	\		
		Spectral stitching method [77]	10,000 με/ ± 3.2 με	1 cm	1170 m		
		Spectral cursor technology [78]	10,856 με/ 18.7 nε/√Hz	\	10 m		
	Hardware calibration or algorithm compensation	Time-scale factor correction method [79]	\	0.17 mm	155 m	Corrects sweep nonlinearity	Performance degrades with field drift
		Periodic phase noise estimation with de-slope filtering [80]	\	Sub- millimeter	8 km		
	Digital signal processing	Wavelet noise reduction algorithm [81]	2.3 με	\	300 m	Improves reconstruction quality	Tuning dependence
		Three-dimensional image noise reduction idea [82]	\	\	50 m		
		Block matching and principal component analysis [83]	0.31 με	5 cm	\		
		Kalman prediction algorithm [84]	0.5 °C/±5 με	\	50 m		

**Table 3:** The performance of φ-OTDR demodulation technology for UWFBG-based DFOS networks.

Direction	Method	Concrete Scheme	Performance Breakthrough	Remaining Gaps
SNR enhancement	Phase noise compensation	Auxiliary interferometer [89]	Equivalent phase noise floor: −71.2 dB re rad/√Hz	Drift sensitivity; added reference complexity
	Advanced modulation and coding	Dual-pulse detection scheme [90]	SNR to 45.92 dB at 0.1 Hz/ 43.33 dB at 30 kHz	Decoding cost; sensitive to nonideal modulation
		Golay coding modulation technique [91]	SNR to 62.2 dB at 100 Hz	
	Polarization fading suppression	Polarization diversity scheme [92]	Stable SNR exceeding 30 dB	Additional channels/hardware
Dynamic range optimization	Multi-wavelength and optical unwrapping	Dual-wavelength probing scheme [93]	Unwrap the phase beyond the conventional π-constraint	Wavelength stability needed; processing and parameter tuning required
		Chirped-pulse interrogation schemes [94]	Expand the measurable range of dynamic strains	
	Algorithmic phase unwrapping	Differential-unwrapping-integral algorithm [95]	Mitigate projection errors	Algorithm complexity; scalability and real-time performance constraints
		Least squares linear programming phase unwrapping algorithm [96]	Large dynamic strains ranging from 13.8 to 44.6 με	
	Deep learning-enhanced unwrapping	Deep neural network-based 2-D phase unwrapping method [97]	Predict and correct wrapping errors in real time	Training data and interpretability issues

**Table 4:** The performance of OTDR demodulation technology for UWFBG-Based DFOS Networks.

Method	Concrete Scheme	Spatial Resolution	Length	Speed	Typical Benefits	Remaining Gaps
Hybrid multiplexing integrates	TDM–WDM + SOA [99]	0.2 m	200 m	\	Increases sensor capacity	Channel discrimination
	TDM–WDM + high-speed imaging spectral demodulation method [100,101]	2 m	10 km	\		
Aliased spectrum demodulation	Segmented spectrum reconstruction + relaxed pulse width [103]	10 cm	2 km	\	Enables denser sensing	Aliasing artifacts
	One-dimensional deep convolutional neural network [104]	10 cm	\	\		
Dispersion-based method	Dispersion compensation fiber [105]	\	\	100 kHz	Speeds up readout	Temperature sensitivity; accuracy limits
	Chromatic dispersion + ultra weak FBGs [106]	1 m	2 km	20 kHz		
The edge-filtering technique	High birefringence fiber loop mirror [107]	15 m	\	200 kHz	Simple and fast	Limited linear range
Advancing swept laser technology	FDML laser + TDM–WDM [108]	5 m	1 km	100 kHz	Faster sweep	Stability and calibration burden

**Table 5:** The performance of OFDR demodulation technology for UWFBG-based DFOS networks.

Direction	Method	Spatial Resolution	Accuracy	Speed	Typical Benefits	Remaining Gaps
Demodulation accuracy optimization	Auxiliary optical path + direct spectral peak-finding algorithm [110]	1.28 mm	\	38 Hz	Enhances demodulation accuracy	Peak ambiguity under noise
	Auxiliary interferometer + phase-shifted FBGs [111]	\	0.06 °C	\	Higher sensitivity	Interferometer drift
Demodulation speed enhancement	Weighted sliding window Fourier transform + logical activation function threshold processing [112]	0.3 mm	11.6 pm	Speed to 24 times	Faster updates with acceptable precision	Parameter- sensitive
	FPGA-based two-dimensional FFT + frequency domain cross-correlation algorithm [113]	6.4 mm	867 m	24 Hz	Real-time processing for large-scale arrays	Reduced flexibility/portability

## Data Availability

The original contributions presented in this study are included in the article. Further inquiries can be directed to the corresponding author.

## Abbreviations

The following abbreviations are used in this manuscript:

DFOS	Distributed fiber optic sensing
UWFBG	Ultra-weak fiber Bragg grating
SNR	Signal-to-noise ratio
OTDR	Optical time domain reflectometry
DTS	Distributed temperature sensing
RDTS	Raman distributed temperature sensing
FBG	Fiber Bragg grating
OFDR	Optical frequency domain reflectometry
ROTDR	Raman optical time domain reflectometry
ASE	Amplified spontaneous emission
WTMM	Wavelet transform modulus maxima
1DDCNN	One-dimensional denoising convolutional neural network
BOTDR	Brillouin optical time domain reflectometry
EDFA	Erbium-doped fiber amplifier
BFS	Brillouin frequency shift
DCS-BOTDR	Differential cross-spectrum Brillouin optical time domain reflection
RMSE	Root mean square error
SAID	Spatial adaptive image denoising
BGS	Brillouin gain spectrum
EOM	Electro-optic modulator
I-OTDR	Intensity-based optical time domain reflectometry
φ-OTDR	Phase-sensitive optical time domain reflectometer
LMS	Least mean square
MBCP	Multi-band chirped pulse
EMD-TFPF	Empirical mode decomposition and time–frequency peak filtering
RBS	Rayleigh backscattering signal
PPNE	Periodic phase noise estimation
LO	Local oscillator
ls-LPPU	Least squares linear programming phase unwrapping
TDM	Time division multiplexing
WDM	Wavelength division multiplexing
SDM	Space division multiplexing
SOA	Semiconductor optical amplifier
DCF	Dispersion compensating fiber
HiBi-FLM	High birefringence fiber loop mirror
FRM	Faraday rotating mirror
